# Health Outcomes of Exposure to Biological and Chemical Components of Inhalable and Respirable Particulate Matter 

**DOI:** 10.3390/ijerph13060592

**Published:** 2016-06-14

**Authors:** Oyewale Mayowa Morakinyo, Matlou Ingrid Mokgobu, Murembiwa Stanley Mukhola, Raymond Paul Hunter

**Affiliations:** Department of Environmental Health, Faculty of Science, Tshwane University of Technology, Private Bag X680, Pretoria 0001, South Africa; Mokgobumi@tut.ac.za (M.I.M.); MukholaMS@tut.ac.za (M.S.M.); paul.hunter@uea.ac.uk (R.P.H.)

**Keywords:** particulate matter, biological composition, chemical composition, health outcomes, disease burden

## Abstract

Particulate matter (PM) is a key indicator of air pollution and a significant risk factor for adverse health outcomes in humans. PM is not a self-contained pollutant but a mixture of different compounds including chemical and biological fractions. While several reviews have focused on the chemical components of PM and associated health effects, there is a dearth of review studies that holistically examine the role of biological and chemical components of inhalable and respirable PM in disease causation. A literature search using various search engines and (or) keywords was done. Articles selected for review were chosen following predefined criteria, to extract and analyze data. The results show that the biological and chemical components of inhalable and respirable PM play a significant role in the burden of health effects attributed to PM. These health outcomes include low birth weight, emergency room visit, hospital admission, respiratory and pulmonary diseases, cardiovascular disease, cancer, non-communicable diseases, and premature death, among others. This review justifies the importance of each or synergistic effects of the biological and chemical constituents of PM on health. It also provides information that informs policy on the establishment of exposure limits for PM composition metrics rather than the existing exposure limits of the total mass of PM. This will allow for more effective management strategies for improving outdoor air quality.

## 1. Introduction

Clean air is a requirement for life and healthy living, a fundamental human right. An adult requires between 10,000 and 20,000 liters of air per day for survival [1]. Staying and remaining healthy requires constant breathing in of clean and safe air. The World Health Organization (WHO) reported that an estimated 1.3 million deaths are ascribed to urban outdoor air pollution annually [2]. The reason being that the air we breathe often contains particulate matter (PM) of varied sizes and compositions. PM is introduced into the atmosphere during air pollution process, and its presence in the atmosphere may be injurious to humans, living organisms, and the natural environment [3,4]. PM according to the WHO, affects more people than any other pollutant [2].

PM is not a self-contained pollutant but a mixture of several pollutants distributed differently at various sizes. The United State Environmental Protection Agency (USEPA) defined PM as ‘‘a complex mixture of extremely small particles and gases and includes acids, organic chemicals, metals, soils and dust’’ [5]. The size of a PM varies from a few nanometers (nm) to tens micrometers (μm) [6]. It is usually expressed by mass concentration in terms of PM_0.1_ (aerodynamic diameter less than 0.1 μm), PM_2.5_, (aerodynamic diameter less than 2.5 μm) or PM_10_ (aerodynamic diameter less than 10 μm) [7]. PM_10_ (coarse or “inhalable” particles) can infiltrate into the human respiratory system, PM_2.5_ (fine or “respirable” particles) can penetrate into the gas-exchange region of the lung), while PM_0.1_ (ultrafine particles) provides a large surface area, with degrees of lung permeation [6]. Inhalable PM is a fraction of PM that is hazardous when deposited anywhere in the respiratory tract. Whereas, respirable PM are fractions of inhaled particles that are capable of passing beyond the human larynx and ciliated airways [8]. The size, mass and surface area of a PM are directly linked to its potential for causing health problems. 

Though it may be apt to cluster PM as particulates, their sources, spread and effects may be highly varied [9]. These particles can originate from natural sources, such as biological particles (pollen, fungal spores, *etc*.), fine soil particles, fine marine salts, wildfire smoke particles and volcanic ash, among other things [9]. Some are from industrial combustion processes, vehicle emissions, domestic heating and cooking, burning of waste crop residues, land clearing, and fire control activities. Others are from the reaction of gaseous precursors (secondary particles) [9] emitted at distant locations and transported by atmospheric processes. The presence of PM from different sources varies with time, season, location and climate, thus resulting in spatial and season-dependent variations in concentration, characteristics, and toxicity [10,11].

**Figure 1:**
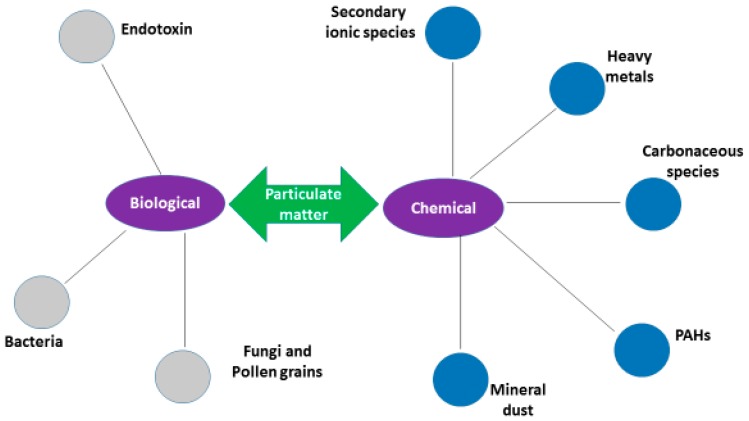
Biological and chemical components of particulate matter.

PM contains different physical characteristics (particle size and number, total surface area, and electrostatic properties) [12], biological and chemical components (Figure 1) [7]. The biological components, also known as bioaerosols, are a mixture of viable and non-viable microorganisms as well as other types of biomass suspended in the air with their sizes ranging from <0.1 µm to ≤100 µm [13]. They tend to attach in a coarser particulate fraction, however, fungal spores, fragmented pollen, and non-agglomerated bacteria are also present in the fine fraction [14].

The chemical components of PM include mineral matter (oxides of aluminum, calcium, silicon, titanium, iron, magnesium, manganese, sodium and potassium), organic matter, elemental carbon, secondary inorganic aerosol, sea salt and trace elements [15]. Among these components, secondary inorganic aerosols (sulfate, nitrate, and ammonium) and carbonaceous particles are of great concern, as they are crucial factors controlling the degree of acidity and toxicity of the PM [16].

Although exposure to PM has been implicated in the causation of diverse health outcomes [17,18,19,20,21,22,23], not much has been reported on the role each or mixture of the components of PM plays in the occurrence of adverse health outcomes. The exact components of PM that effect disease causation and the modalities involved are relatively unknown. Studies to determine the components of PM that contribute to airway inflammation and irritation have however been attempted [24,25]. Describing the importance of the effects of each or mixtures of these components of PM on human health is of public health significance.

The aim of this review paper is to summarize the global evidence of the effects of the biological and chemical components of inhalable and respirable PM on health, and to recommend future focus areas for research and policy. 

## 2. Methods

The authors conducted a scientific review of accessible literature published over the last 30 years. Our main objective was to provide evidence of the role of biological and chemical components of PM in the causation of adverse health effects in humans. We commenced a PubMed database search using the MESH terms “PM”, “particulate matter”, “air pollution”, “ultrafine particles”, “fine particles”, “coarse particles”, “PM_10_”, “PM_2.5_”, “PM_0.1_”, “Bioaerosols in PM”, “Bacteria in PM”, “Endotoxin in PM”, “Fungi and pollens in PM”, “trace elements in PM”, “secondary inorganic species in PM”, “Polycyclic aromatic hydrocarbon in PM”, “Inorganic mineral dust in PM”, “Elemental carbon in PM”, “Organic carbon in PM”, and “Black carbon in PM”, “Health effects”. Literature was also sourced from other scientific databases including ProQuest and Science Direct online database search. Articles were selected and agreed upon by the authors based on relevance and usefulness.

## 3. Particulate Matter-Associated Bioaerosols

Airborne PM comprises a substantial fraction of biological components [26]. Bioaerosols, which originate from biological sources and mostly associated with PM, are solid or liquid particles that are present in the gaseous medium [27]. Bioaerosols are generally planted pollen, microorganisms (fungi, bacteria, viruses) or organic compounds that evolve from microbes (endotoxins, metabolites, toxins and other microbial fragments) [28]. Stetzenbach [29], opined that about 5% to 34% of air pollutants is composed of bioaerosols. Bioaerosols attached to PM can exist either as non-viable biomolecules (e.g., antigenic compounds, dead skin cells, dander, plant and insect debris), non-viable microorganisms or as viable microorganisms [30].

In 2002, [31] termed bioaerosols to differ in mass and structure and are subject to the source, aerosolisation, and environmental conditions prominent at the site. Most bioaerosols are of the respirable size of 0.003 μm for viruses [32], 0.25 to 20 μm for bacteria [33], 17 to 58 μm for plant pollens [34], and 1 to 30 μm for fungi [35]. Bauer *et al.* [36] reported that Fungi accounted for up to ~10% of organic carbon, and ~5% of PM_10_ at urban and suburban locations and abundant in a coarser particulate fraction. However, Meklin and colleagues were of the opinion that fungal spores, fragmented pollen, and non-agglomerated bacteria are also present in a fine fraction of PM [13]. Other researchers reported that biological sources of PM accounted for between 5% and 10% of the urban and rural aerosol composition [37,38].

Bioaerosols can attach to PM from varied sources (e.g., traffic, industry, soil), have its aerodynamic and antigenic properties altered, and thus aiding its penetration into deeper regions of the lung [39]. For instance, inhalation of whole pollen (>10 µm) cannot reach the small airways, however, pollen allergens present in PM_2.5_ can easily penetrate the small airways of the lung [40]. Thus, the discrete effects of bioaerosols and PM, as well as their combined effects, can exacerbate respiratory allergy and other pulmonary diseases. A study done in the Cincinnati area revealed that high concentration of PM_10_ was synergistic with the airborne pollen concentration levels for envisaging daily asthma visits [41].

Adhikari and colleagues also reported that the combined effect of bioaerosols and PM can aggravate respiratory allergy and other pulmonary diseases in human [42]. It could trigger allergic, toxic, and infectious responses in exposed individuals [43,44,45]. Symptoms in exposed individuals can include coughing, wheezing, runny nose, irritated eyes or throat, skin rash, diarrhea, aggravation of asthma, headache, and fatigue. Immunological reactions include asthma, allergic rhinitis, and hypersensitivity pneumonitis [43,44,45]. Table 1 summarizes the available information on the types of study and biological pollutants analyzed (either singly or in combination with PM), study population and location, observed health effects, and the details of cited references.

### 3.1. Particulate Matter-Associated Endotoxins

Endotoxin, an important biological component of PM is ubiquitous in the environment and is a key structural constituent of the outward membrane of Gram-negative bacteria [46]. Endotoxin is reported to be present in ambient PM at low levels. Some researchers reported that the endotoxin concentration in inhalable particles was 3–10 times higher than that in respirable particles [47,48]. However, other researchers assert that airborne endotoxins are considerably linked with PM_2.5_ [49,50,51] and are deposited in the lungs after inhalation [52].

Exposure to endotoxin has been reported to cause and trigger asthma and wheezing occurrence in children and adults [53,54]. Liebers *et al*. [55] and Rabinovitch *et al*. [56], implicated endotoxin in the weakening of the functioning of the lung, and the pathogenesis of pulmonary diseases such as organic dust lung diseases [57], chronic obstructive pulmonary diseases (COPD) [44], and acute lung injury [58]. Different studies have pointed out the role of endotoxin in PM toxicity both *in vitro* [59,60] and *in vivo* [61]. Inhalation of endotoxins together with other airborne pollutants such as PM, fungi, allergens, and ozone, have been documented to increase the susceptibility to and severity of an immune response, and can lead to other adverse health effects [62,63,64].

Therefore, it can be inferred that the airborne biological particles, a fraction of which is endotoxin, plays a significant role in the proinflammatory response. This is consistent with other previous findings that have been reported [65,66]. However, the actual role of endotoxin in inducing proinflammatory response is not well understood [67].

### 3.2. Particulate Matter-Associated Bacteria

Airborne bacteria are one of the main components of airborne biological particles in natural and urban environment. This is in addition to being key components of outdoor and indoor aerosols [68,69,70]. Contemporary knowledge of the distribution of bacteria in the atmosphere is quite inadequate. This is because most bioaerosols studies relied solely on culture-based techniques [71,72] or accounted only for the whole fraction of the PM [73]. However, recently, culture-independent techniques have been used in the study of bioaerosols associated with small size particles [74] and the characterisation of the spatial or temporal variations of bioaerosols in urban environments [69,75]. High concentrations of airborne bacteria can have major effects on human health as pathogens or triggers of allergic asthma and seasonal allergies [68]. 

### 3.3. Particulate Matter-Associated Fungi and Pollen Grains

Dominant biological component of airborne coarse particles are fungal spores [76]. They are produced during the life cycle of a fungus, and whose size range between 2 and 10 μm [77]. They originate from sources, such as plants, animals, soil and human activities. Kendrick [78] asserts that there are over 100,000 fungal species whose spores may become airborne. Earlier studies stated that PM may possibly bind with airborne pollen [79] and fungal spores [77] thus altering their morphology. Womiloju *et al*. [38], reported that cell materials of fungi and pollen could contribute 4%–11% of the total PM_2.5_ mass and 12%–22% of organic carbon in fine particulate matter. 

In a study conducted in Cincinnati in the United State on the correlation of ambient inhalable bioaerosols with PM and ozone, the predominant airborne fungi and their corresponding percentages relative to the total airborne fungal load found during the entire sampling period were: *Aspergillus/Penicillium* group (41.6%), *Cladosporium* (28.4%), *Ascospores* (10.6%), *Basidiospores* (9.8%), smut spores (2.6%), *Alternaria* (1.4%), *Epicoccum* (0.7%), and rust spores (0.2%) [42]. Bauer and colleagues in their study that focused on knowing the significant contributions of fungal spores to the organic carbon and to the aerosol mass balance of the urban atmospheric aerosol discovered that fungal spores are the main constituents of coarse organic PM in the summer season [36].

Fungal spores are recognized risk factor for adverse health effects, such as inflammatory responses associated with allergies and asthma [80,82,83]. Among different bioaerosol components, airborne fungi and pollen grains are associated with respiratory allergic diseases and asthma [84,85]. Various studies around the world have investigated the ambient airborne fungi and pollen in relation to respiratory allergies [81,86]. 

## 4. Chemicals in Airborne Particulate Matter

Chemical components of PM are highly varied. They can generally be classified as carbonaceous fractions including organic carbon, elemental carbon, carbonate carbon and inorganic components consisting of crustal elements, trace metals, and ionic species. Each of these components typically contributes about 10%–30% of the total PM mass load [87,88].

Chemical constituents of PM can trigger allergic and asthmatic reactions caused by exposure to bioaerosols. Epidemiologic studies examining sources and composition of PM have identified several definite components, including elemental carbon, organic carbon, and nitrates as associated with increased risk for cardiovascular and respiratory hospital admissions [89,90] and mortality [91]. Elemental components of PM_2.5_, including Ni, Zn, Si, Al, V, Cr, As, and Br, have also been linked with increased cardiovascular and respiratory hospital admissions [89,92], increased mortality [93], and lower birth weight [94].

Many studies have examined the association between adverse health effects and the toxicity of the diverse chemical components of PM [95] and among others the role that transition metals [60,96] and organic species (polycyclic aromatic hydrocarbons and quinones) [97,98] played in PM toxicity. Findings from toxicological studies reported that organic compounds and transition metals present in PM_2.5_ may be significant due to their ability to stimulate inflammation with subsequent respiratory and cardiovascular effects [99]. However, the United Kingdom Department of Health Committee on the medical effects of air pollution [100] affirmed that no known single chemical substance in PM is of sufficient toxicity to cause the observed magnitude of health effects. 

Studies that demonstrate the role of chemical components of inhalable and respirable PM in the causation of adverse health effects are presented in Table 2. Only studies written in English and with information on the types of study and chemical component were analyzed (in combination with PM), and study population and location, and observed health effects were examined. The reference list of the reviewed articles was also included. 

### 4.1. Particulate Matter-Associated Trace Metals

Present in virtually every aerosol size fractions of airborne PM are trace metals [120]. Different metals such as Cd, Cr, Cu, Mn, Ni, Pb, V, and Zn have been reported to be widely distributed in PM [121]. Their existence in PM originates from the combustion of fossil fuel, incineration, high-temperature metal processing and from soil dust [30]. Humans can be exposed to airborne metals in PM through inhalation of fine particulates, dermal contact and ingestion through deposition of particulates into foods and drinks [122].

The combined risk of exposure to multi-elements in fine particulate via the inhalation route has been reported to exceed acceptable limit [101]. Several epidemiological studies have revealed that exposure to particulate bound trace metals can exacerbate adverse human health effects [102,123,124]. Cu, Zn, and V have been implicated in the causation of diverse cardiovascular effects, together with increased expression of different cytokines and stress proteins, reduction in spontaneous beat rate, vasoconstriction, and vasodilation [125,126]. Metal-bound fine respirable particles have similarly been known to cause lung or cardiopulmonary injuries [127]. Exposure to the elevated amount of lead and manganese can trigger neurological and haematological effects in children [128] while exposure to As, Cd, Cr, and Ni compounds have been linked to the occurrence of cancer in human [129]. Moreover, Vanadium compounds, mostly vanadium pentoxide are associated with health effects of the human respiratory tract [130].

Remarkably, the effects resulting from exposure to metal-bound PM may be triggered by a complex interaction between different metals. Campen and colleagues [131], reported that nickel and vanadium may interact synergistically to effect instant and delayed cardiovascular effects. For instance, exposure to nickel in PM was reported to cause delayed bradycardia, hypothermia and arrhythmogenesis effects: however, vanadium alone did not cause any significantly delayed effects, but enhanced the effects of nickel [131].

Researchers in their studies assert that the resultant health outcomes associated with exposure to metals in PM start from the inhalation of these particles during breathing, followed by settling of the particles in the human respiratory system. Moreover, ultrafine particles less than 1 µm can travel deeper into alveolar region of lungs where they mix with the lung fluid [132,133], and can be absorbed into human physiological systems thus exerting an adverse toxic effect. Though quite a number of studies have specified that metals are among the contributory components in PM-induced effects, the relationship may not be direct.

### 4.2. Particulate Matter-Associated Polycyclic Aromatic Hydrocarbons

Polycyclic aromatic hydrocarbons (PAHs) are a large group of abundant, persistent semi-volatile organic compounds comprising of two or more bonded aromatic rings structured in various configurations [134,135,136]. They are formed from the incomplete combustion and pyrolysis of organic materials such as coal, oil, gas, and wood [137,138] and are released into the environment from natural (e.g., volcanic eruptions and forest fires) and anthropogenic sources (coal, oil and gas burning facilities, motor vehicles, waste incineration and industrial activities) [139].

PAHs differ in their molecular weight and structure [135]. Low molecular weight PAHs appears to be more available in the vapor phase while higher molecular weight PAHs are mostly associated with particulates [140]. For instance, atmospheric PAHs with 2–4 aromatic rings are assigned between PM and gas phase, whereas the ones with high molecular weight consisting of more (4–6) aromatic rings are mostly in the fine (PM_2.5_) fraction of particulate phases [141,142]. The behavior of PAHs in the atmosphere is contingent upon complex physicochemical reactions, interactions with other pollutants, photochemical transformations, and dry and wet deposition [143].

Moreover, PAHs in the ambient air can be attached to airborne particulate matter owing to atmospheric conditions, the nature of the aerosol, and the properties of individual PAHs [144]. Recognized carcinogenic PAHs have been found to be mostly associated with PM [145,146]. Some researchers also indicated that PAHs have their highest concentration in the respirable size range of airborne PM [147,148].

Akyuz and Cabuk in their study on particulate-associated PAHs in the atmospheric environment of Zonguldak in Turkey observed that the predominant PAHs determined in PM_2.5_ were pyrene, fluoranthene, benzo(a)anthracene, chrysene, benzo(b)fluoranthene and benzo(a)pyrene [149]. The total concentrations of PAHs were up to 464.0 ng·m^−3^ in fine and 28.0 ng·m^−3^ in a coarse fraction in winter, and up to 22.9 and 3.0 ng·m^−3^ in summer months respectively [149]. Higher concentration of PAHs was detected in fine particulates during winter as a result of higher adsorption of PAHs on fine particulates owing to their large surface area per unit mass. Approximately 93.3% and 84.0% of total PAHs in winter and summer months respectively were determined in PM_2.5_, penetrating the pulmonary alveoli and inducing adverse effects in human [149]. Studies done elsewhere on airborne particulates indicated that PAHs immersed in the PM may trigger adverse health effects [150,151]. 

Concern about exposure to PAHs in PM has been on the rise over the years due to their persistence, bioaccumulation, and carcinogenic, and mutagenic effects [152]. Most PAHs analyzed by the International Agency for Research on Cancer showed that benzo(a)anthracene, benzo(a)pyrene and dibenzo(a,h)anthracene were classified as probably carcinogenic to humans; while, naphthalene, benzo(b)fluoranthene and benzo(k)fluoranthene were classified as possibly carcinogenic to humans [149]. The most intoxicating PAH carcinogens have been identified to include benzo(a) anthracene, benzo(a)pyrene, and dibenz(ah)anthracene [134,153]. 

Short-term exposure to PAHs could impair lung function in asthmatics and thrombotic effects in people affected by coronary heart disease [154]. Mixtures of PAHs are known to cause skin irritation and inflammation [155]. Human cancer causes of skin, lungs, and bladder have always been associated with PAHs [103]. Exposure to PAHs may also induce cataracts and result in kidney, liver damage and jaundice [156]. Breathing or swallowing large amounts of naphthalene can cause the breakdown of red blood cells [157]. Moreover, PAHs can exert harmful effects on reproduction and immune function [158,159]. Long-term exposure to PAHs is alleged to raise the risks of cell damage via gene mutation and cardiopulmonary mortality [160].

The U.S.’s Center for Children’s Environmental Health (CCEH) demonstrated that exposure to PAH pollution during pregnancy is correlated with adverse birth outcomes such as low birth weight, premature delivery, and delayed child development [104]. High prenatal exposure to PAHs is also associated with low intelligent quotient at age three, increased behavioral problems at ages six to eight, and childhood asthma [105,161]. A study on childhood leukemia established a positive association between exposure to benzene and the risk of childhood leukemia [148]. Exposure to air pollution containing ultrafine particles and high levels of benzene were associated with increased oxidative DNA damage [162]. However, though PAHs are known for their carcinogenicity characteristics, there is still no threshold for a dose-response relationship established for PAHs [163].

### 4.3. Particulate Matter-Associated Inorganic Water Soluble Ionic Species

One of the chemical constituents of airborne PM are water soluble anions (e.g., NO_3_^–^, SO_4_^2–^, Cl^–^, F^–^, NO_2_^–^, Br^–^) and cations (e.g., NH_4_^+^, Na^+^, K^+^, Ca^2+^, Mg^2+^) [164]. Several studies have revealed the concentration of ionic species in PM [165,166]. Zhao and Gao [167] reported that PM_1.8_ made up 68% of PM_10_ mass concentrations, and water-soluble ions accounted for more than 50% of PM_1.8_ mass concentrations.

Other researchers assert that in addition to organic species, sulfate, nitrate and ammonium ions were the dominant constituents of water-soluble ions in PM [165,168,169,170]. Ying and Kleeman [171] in their study conducted in the South Coast Air Basin, California, USA, reported that 80% of nitrate and ammonium in PM_2.5_ was formed from a precursor gas. However, Han *et al.* [172] showed that ionic constituents accounted for 35%–60% of PM_2.5_ mass in industrial and urban cities of Korea. As a key inorganic constituent of fine aerosols, sulfate, nitrate and ammonium are also linked with atmospheric visibility degradation, the acidity of precipitations and conductivity, and adverse human health effects [173].

Lippmann and Thurston in their study found statistically significant associations between sulfate and respiratory outcomes [174]. Other studies in Canada and the US reported the effects of sulfate on human health: on mortality [106,175], on hospital admissions [107], on respiratory health of children [176] and on emergency room visits [108]. However, a study conducted in The Netherlands reported no association with acute respiratory symptoms in children [177]. Although an association between sulfate and respiratory illnesses seems well documented, a related cause-effect relationship may be flawed by the strong correlation with PM_2.5_ and acidity.

Furthermore, an increase in the number of persons becoming ill has been reported when airborne concentrations of PM_2.5_ and PM_2.5_ SO_4_^2−^ increases [178]. Reported illnesses include respiratory problems, changes in heart rhythms, heart attacks, and severe respiratory and heart malfunctions leading to death. Dockery *et al.,* [175] in the Harvard six cities study, found that increases in PM_2.5_ mass and PM_2.5_ SO_4_^2−^ are associated with increases in death rates. This includes deaths from all causes and death precisely from respiratory and heart problems, as well as from lung cancer.

### 4.4. Particulate Matter-Associated Inorganic Mineral Dust

Over the past decades, fewer studies existed that ascertained the correlations between inorganic mineral dust and health effects. In Europe, Perez *et al.* [179] brought inorganic mineral dust, the often overlooked component of PM, to the lead of public health with their study assessing the relationship between exposure to PM_10_ from Saharan dust and daily mortality. This study revealed that daily mortality in Barcelona increased by 8.4% (per increase of 10 µg/m^3^ of PM_10_) on Saharan dust days compared to 1.4% on non-Saharan dust days but no increased risk was observed with PM_2.5_ [117]. An increase in daily emergency room visits for bronchitis for each 100 µg/m^3^ increase in PM_10_ was reported by Hefflin and colleagues in 1994 during a study of the effects of dust storms in Washington State [180].

Moreover, Jimenez *et al*. [181] reported a more pronounced health effects among the elderly (>75 years) from exposure to PM_10_ on dust days in Madrid. The percentage of days in their study, 11.9% of non-dust days *versus* 41.3% on dust days, exceeded the WHO daily health-protection levels for PM_10_ (mean 50 µg/m^3^). Mallone *et al.* [182] also reported an increase in mortality for cardiovascular, circulatory and respiratory causes in Rome linked to increases in PM_10_ on Saharan dust days. A study in the Canary Islands established an association between the heart and respiratory mortality, and PM (10 and 2.5) with rates of respiratory mortality increased by 4.9% for each PM_10_ increase of 10 µg/m^3^ [183].

However, not all studies reported an association between far traveled dust and increased rates of morbidity or mortality. Bennet *et al.* [109] in a retrospective study in British Columbia proved that there was no evidence of increased hospitalization for respiratory or cardiovascular illnesses. In addition, there were no ample changes in clinic attendance for pediatric asthma cases in Barbados in relation to short-term increases in dust concentrations from Africa [184].

### 4.5. Particulate Matter-Associated Carbonaceous Species

A sizeable fraction of fine particles (PM_2.5_) constitutes the carbonaceous aerosol; one of its top three components [185]. It accounts for about 40% of PM_2.5_ mass in urban air [186], 60% of PM_2.5_ in the U.S. [187], 20%–40% [188] and 25%–50% [189] to ambient PM_10_ and PM_2.5_ mass respectively.

Carbonaceous species can be grouped into elemental carbon (EC) and organic carbon (OC). EC (occasionally called black carbon) is formed from the incomplete combustion of materials containing carbon, whereas OC can either be released directly into the atmosphere (primary OC) or produced from gas-to-particle reactions (secondary OC) [190]. OC embodies a mixture of hundreds of organic compounds, some of which are mutagenic and/or carcinogenic, such as PAHs, polychlorinated dibenzo-p-dioxins, and dibenzofurans (PCDD/Fs) [191].

## 5. Discussion

Our review, which examined literature on the biological and chemical components of PM provides important insights into the link between exposure to these constituents of PM and health outcomes. It is remarkable that most of the literature reviewed showed the contribution of PM components to observed health effects. This proved that exposure to PM alone does not trigger or cause health response but also its components, which often determine its toxicity. 

Unlike the chemical components of PM, the impact of biogenic aerosols (bioaerosols) on health relating to inhalation of PM has not been well understood. A sizeable portion of airborne PM are bioaerosols accounting for between 5% and 10% of the urban and rural PM composition [36,37,39]. These bioaerosols are mostly fungi, bacteria, endotoxins, plant pollen, and spore material, all of which have the potential to illicit allergic symptoms. One could say that the individual effects of bioaerosols and PM as well as their synergistic effects, can aggravate respiratory allergy and other pulmonary diseases. Findings from our review of literature show that endotoxins are an important component of PM and are associated the progression of airway diseases [43]. Reduced functioning of the lungs, occurrence of asthma and other pulmonary diseases were reported among children and adults who were exposed to an elevated concentration of endotoxins [48,53,54,55,56,57,58]. Exposure to endotoxins on PM_10_ particles in a toxicological study resulted in elicited upregulation of the Platelet-derived growth factor (PDGF) [110]. Elsewhere, PM_10_ with relatively high levels of endotoxin induces proinflammatory cytokine release via an endotoxin-dependent mechanism [63]. Furthermore, fungal spores and pollen grains associated with PM are known risk factor for inflammatory responses such as asthma [80,81,82,83,84,85,86].

In addition, the association between the different chemical components of PM and adverse health effects were reported by several studies [101,103,104,106,108,112,115]. Bell *et al*. [94] reported that increased exposure to metals in PM_2.5_ such as Zn, Al, V, Si, and Ni, resulted in an incidence of low birth weight among pregnant women. Formation of reactive oxygen species and subsequent lung injury, inflammation, and airway hyper responsiveness that resulted in airflow limitation and symptoms of asthma was recorded among healthy volunteers that were exposed to metals in PM in a toxicological study [102]. In a cross-sectional study of non-smoking individuals, an exposure to V, Fe and Ni contents of PM_2.5_ were significantly associated with systolic blood pressure and pulse pressure [17]. 

Moreover, increased hospitalizations for respiratory and cardiac illnesses were recorded among the general population in British Columbia who were exposed to mineral dust in PM_10_ [109]. Burnette *et al.* [107] reported that a 13-μg/m^3^ increase in sulfates coated-PM was associated with a 3.7% increase in respiratory admissions and a 2.8% increase in cardiac admissions for all age groups in Canada. In totality, the studies considered in our review implicated the different biological and chemical constituents of PM in the causation of ill health.

## 6. Conclusions

In summary, findings from studies reviewed in this paper made it clear that though the particulate matter is a complex heterogeneous mix of remarkably small particles and gases that are capable of inducing adverse health effects in humans. Its biological and chemical components are culpable for the different health outcomes observed in humans. This implies that health effects linked to exposure to particulate matter are dependent on the physical properties, and the chemical and biological compositions of the particulate matter. Bringing into bare the components of particulate matter that drive the association between exposure and particulate-induced health outcomes is crucial to public health, and allow for more operative regulatory guidelines that will improve outdoor air pollution and thereby prolong human lives. Moreover, it is only with this information that strategies aimed at effectively managing the menace of particulate matter in the environment, so as to ensure environmental sustainability, can be developed. It will also provide evidence that will inform policy in the establishment of standard guidelines for the biological and chemical constituents of particulate matter rather than the total mass of ambient particulate matter. It is worth mentioning that there were no or very few studies reported from the low income and middle-income countries. With the upsurge in human population, industrialization, urbanization, modernisation and its attendant increase in vehicular emissions, studies on the health outcomes of exposure to inhalable and respirable particulate matter in these countries should be given more priority. In addition, more studies are needed to better understand the contribution of the combination of the biological and the chemical components of particulate matter to documented health-end points, which have not been fully understood.

## Figures and Tables

**Table 1:** Summary of epidemiological and toxicological studies on health effects of exposure to biological components of PM.

Study	Type of Study	Study Population	Study Location	Pollutant Analyzed	Health Outcome
Schwartz *et al.* [44]	Cross-sectional	Grain handlers and postal workers	Iowa City	Endotoxin and grain dust	Concentration of endotoxin in the may be important in the development of grain dust-induced lung disease.
Targonski *et al*. [45]	Cross-sectional	5- to 34-year-olds in the general population 1985-1989	Chicago	Ambient aeroallergen	The odds of a death caused by asthma occurring on days with mold spore counts of 1000 spores per cubic meter or greater was 2.16 times higher (95% CI = 1.31–3.56, *p* = 0.003) than on days on which mold spore counts were less than 1000 spores per cubic meter.
Bolte *et al.* [54]	Cohort		Munich and Leipzig, Germany	Endotoxin	High endotoxin levels increased the risk of repeated wheeze (OR = 1.52; CI = 1.08–2.14).
Loh *et al.* [57]	Cross-sectional	18 healthy non-atopic human subjects		Inhaled endotoxin or lipopolysaccharide (LPS)	Myeloperoxidase, human neutrophil elastase and interleukin-8 in sputum sol, showed a trend towards greater increase following 50 μg LPS.
Alexis *et al.* [61]	Toxicological	9 Healthy subjects	Chapel Hill, NC	PM_2.5–10_, biologic material on PM_2.5–10_	Induced elevated inflammation; increased eotaxin, and increased phagocytosis.
Cakmak *et al.* [80]	Cross-sectional	Children presented with diagnosed conjunctivitis or rhinitis 1993–1997	Eastern Ontario, Canada	Fungal spores and pollen grains	An increase of 551 basidiomycete’s spores per m^3^, or of 72 ragweed grains per m^3^, was associated with an increase of about 10% in hospital visits for conjunctivitis and rhinitis.
Adhikari *et al*. [81]	Cross-sectional	Adult showing symptoms of type-I respiratory allergy	India	Airborne viable and non-viable fungi	52% of the viable airborne fungi identified were allergenic.

**Table 2:** Summary of epidemiological and toxicological studies on health effects of exposure to chemical components of PM.

Study	Type of Study	Study Population	Study Location	Component Analyzed	Health Outcome
Jacobs *et al*. [17]	Cross-sectional	88 non-smoking individuals	Antwerp, Belgium	PM_2.5_, PAHs, transition metals	Increase of 20.8 μg/m³ in 24-h mean outdoor PM_2.5_ was associated with an increase in pulse pressure of 4.0 mmHg (95% CI = 1.8–6.2); V, Fe and Ni contents of PM_2.5_ were significantly associated with systolic blood pressure and pulse pressure; chrysene-5, 6-dione and benzo(a)pyrene-3,6-dione were significantly associated with increases in systolic blood pressure and pulse pressure.
Osornio-Vargas *et al*. [63]	Toxicological	N/A	N/A	EC, bacteria on PMs	PM_2.5_ and PM_10_ samples caused cytotoxicity; PM_2.5_ induces cytotoxicity *in vitro* through an endotoxin-independent mechanism that is likely mediated by transition metals; PM_10_ with relatively high levels of endotoxin induces proinflammatory cytokine release via an endotoxin-dependent mechanism.
Bell *et al.* [89]	Cross-sectional	General population >64 years 1999–2005	106 U.S. Counties	PM_2.5_, Vanadium, nickel, elemental carbon	Positive association between county-specific estimates of short-term effects of PM_2.5_ on cardiovascular and respiratory hospitalizations and county-specific levels of V, EC, or Ni PM_2.5_ content.
Peng *et al.* [90]	Cross-sectional	General population 2000–2006	119 U.S urban communities	PM_2.5_, sulfate, nitrate, Si, elemental carbon, organic carbon matter, sodium, ammonium ions	Ambient levels of elemental carbon and organic carbon matter are associated with risks of emergency hospitalization.
Ostro *et al.* [91]	Cross-sectional	General population	Six California counties	PM_2.5_ mass and components, including elemental and organic carbon (EC and OC), nitrates, sulfates, and various metal	PM_2.5_ mass and several constituents were associated with multiple mortality categories, especially cardiovascular death.
Zanobetti *et al.* [92]	Cross-sectional	General population 2000–2003	US communities	PM_2.5_, elemental composition, ionic species	For a 10 μg/m^3^ increase in 2-day averaged PM_2.5_ concentration, there was an increase of 1.89% in CVD, 2.74% (95% CI: 1.30–4.2) in diabetes, and 2.07% (95% CI: 1.20–2.95) in respiratory admissions; PM_2.5_ mass was higher in Ni, As, and Cr, as well as Br and OC significantly increased its effect on hospital admissions.
Bell *et al.* [94]	Cross-sectional		3 Connecticut counties and 1 Massachusetts county	PM_2.5_, 50 elements, traffic, road dust/crustal	Increase in exposure was associated with low birthweight for Zn, EC, Si, Al, V, and Ni. Analysis by trimester showed effects of third-trimester exposure to EC, Ni, V, and oil combustion PM_2.5_.
Diaz and Dominguez [101]	Cross-sectional	General population	Mexico	EC of PM_2.5_	High risk of contracting diseases associated with elemental exposure.
Gavett and Koren [102]	Toxicological	Healthy volunteers	NA	Ambient PM, Transition metals	Formation of reactive oxygen species and subsequent lung injury, inflammation, and airway hyper responsiveness leading to airflow limitation and symptoms of asthma.
Boffetta *et al.* [103]	Cross-sectional	Industrial workers		PAHs and nitro-PAHs	Risk of lung, skin, and bladder cancer.
Perera *et al.* [104]	Cross-sectional	867 mothers and 822 newborns	Northern Manhattan, The World Trade Center Area, Poland, and China	PM, PAH, benzo( *a*)pyrene	Fetus may be 10-fold more susceptible to DNA damage than the mother and that in utero exposure to PAH may disproportionately increase carcinogenic risk.
Edwards *et al.* [105]	Cohort study	Pregnant, healthy, non-smoking women	Krakow, Poland	PAH	Prenatal exposure to PAH was associated with decreased Raven Colored Progressive Matrices (RCPM) scores at age 5.
Pope *et al.* [106]	Cross-sectional	General population 1980–1989	U.S.	PM, Sulfate	PM was associated with cardiopulmonary and lung cancer mortality; Increased mortality is associated with sulfate and PM_2.5_ at levels commonly found in U.S. cities.
Burnett *et al.* [107]	Cross-sectional	General population 1983–1988	Ontario, Canada	Sulfate	A 13 μg/m^3^ increase in sulfates was associated with a 3.7% increase in respiratory admissions and a 2.8% increase in cardiac admissions for all age groups.
Delfino *et al.* [108]	Cross-sectional	Patients with respiratory illnesses 1992–1993	Montreal, Quebec	PM_2.5_, PM_10_, O_3_, SO_4_^2−^	1-h maximum O_3_, PM_10_, PM_2.5_, and SO_4_^2−^ were all positively associated with respiratory visits for patients over 64 yrs. of age.
Bennet *et al.* [109]	Cross-sectional	General population 1997–1999	Vancouver region of British Columbia, Canada	PM_10_, Desert Dust	Additional one or two hospitalizations per 100,000 population for respiratory and cardiac illnesses.
Bonner *et al.* [110]	Toxicological study	General population	Mexico city	Endotoxins, elemental contents of PM_10_	PM_10_ induce expression of the PDGF a-receptor subtype on rat pulmonary myofibroblasts; endotoxin and metal components of PM_10_ stimulate IL-1b release. Endotoxin on PM_10_ particles elicited upregulation of the PDGF receptor.
Dockery *et al.* [111]	Cross-sectional	ICD Patients	Boston	PM_2.5_, BC, sulfate	Ventricular tachyarrhythmias.
Frampton *et al*. [112]	Cross-sectional	General population	Utah valley	Metal content of PM_10_	Cytotoxicity, induced expression of interleukin-6 and -8.
Ghio *et al*. [113]	Toxicological	38 Healthy volunteers	North Carolina	Ambient particles	Mild inflammation in the lower respiratory tract, and increased concentration of blood fibrinogen.
Hsu *et al*. [114]	Cross-sectional	Elderly patients	New York City	PM_2.5_, PM_10,_ , Elemental carbon (EC), K, Ni, Ca, Fe, Al, Si, Se, V, Zn	Cardiopulmonary function parameters.
Lall *et al.* [115]	Cross-sectional	Medicare hospital Admissions	New York City	EC, Ni, Mn, Si, S	Daily hospital admissions, 2001–2002.
Strickland *et al.* [116]	Cross-sectional	Children 5–17 Years 1993–2004	Atlanta	PM_10_, PM_2.5_, sulfate, EC, OC, water-soluble Metals	Emergency department visits for asthma.
Thurston *et al.* [117]	Cross-sectional	General population 1986–1988	Toronto, Ontario	PM_2.5_, PM_10_, O_3_, (H^+^) and sulfates (SO_4_^−^)	Exposure to O_3_, H^+^, and SO_4_^−^ were significantly associated with respiratory and asthma admissions.
Wellenius *et al.* [118]	Cross-sectional	Hospitalized stroke Patients 1999–2008	Boston area	PM_2.5_, BC, sulfate	Stroke onset.
Zhou *et al*. [119]	Cross-sectional	General population	Detroit, Seattle	PM_2.5_, Al, Fe, K, Na, Ni, S, Si, V, Zn, EC	Mortality: total, cardiovascular, respiratory.

Notes: BC—Black carbon, EC—Elemental carbon, PM—Particulate matter, PAH—Polycyclic aromatic hydrocarbons, PDGF—Platelet-derived growth factor.
